# Supplementary material for: Synthesis of a Small Library of Nature-Inspired Xanthones and Study of Their Antimicrobial Activity
Source: Molecules. 2020 May 21;25(10):2405. doi: 10.3390/molecules25102405 (PMC7287773; doi:10.3390/molecules25102405)
Supplement: Supplementary file 1 [file molecules-25-02405-s001.pdf]

Supplementary material

Synthesis of a Small Library of Nature-Inspired Xanthones and Study of their Antimicrobial Activity

Diana I. S. P. Resende[a,b,†] , Patrícia Pereira-Terra[a,c,†] , Joana Moreira[a,b] , Joana Freitas-Silva[a,c] , Agostinho Lemos[b] , Luís Gales[c,d,e] , Eugénia Pinto[a,f*] , Emília Sousa[a,b*] , Paulo Martins da Costa[a,c] and Madalena M. M. Pinto[a,b]

[a] *CIIMAR - Centro Interdisciplinar de Investigação Marinha e Ambiental, Terminal de Cruzeiros do Porto de Leixões, 4450-208 Matosinhos, Portugal*
[b] *Laboratório de Química Orgânica e Farmacêutica, Faculdade de Farmácia, Universidade do Porto, Rua de Jorge Viterbo Ferreira, 228, 4050-313 Porto, Portugal*
[c] *ICBAS – Instituto de Ciências Biomédicas Abel Salazar, Rua de Jorge Viterbo Ferreira 228, Porto, Portugal*
[d] *i3S – Instituto de Investigação e Inovação em Saúde, Rua Alfredo Allen, 208, Porto, Portugal*
[e] *IBMC – Instituto de Biologia Molecular e Celular Universidade do Porto, Rua Alfredo Allen, 208, Porto, Portugal*
[f] *Laboratório de Microbiologia, Departamento de Ciências Biológicas, Faculdade de Farmácia, Universidade do Porto, Rua de Jorge Viterbo Ferreira, 228, 4050-313 Porto, Portugal*
*Correspondence: esousa@ff.up.pt, epinto@ff.up.pt
† These authors contributed equally to this work.

Spectral data

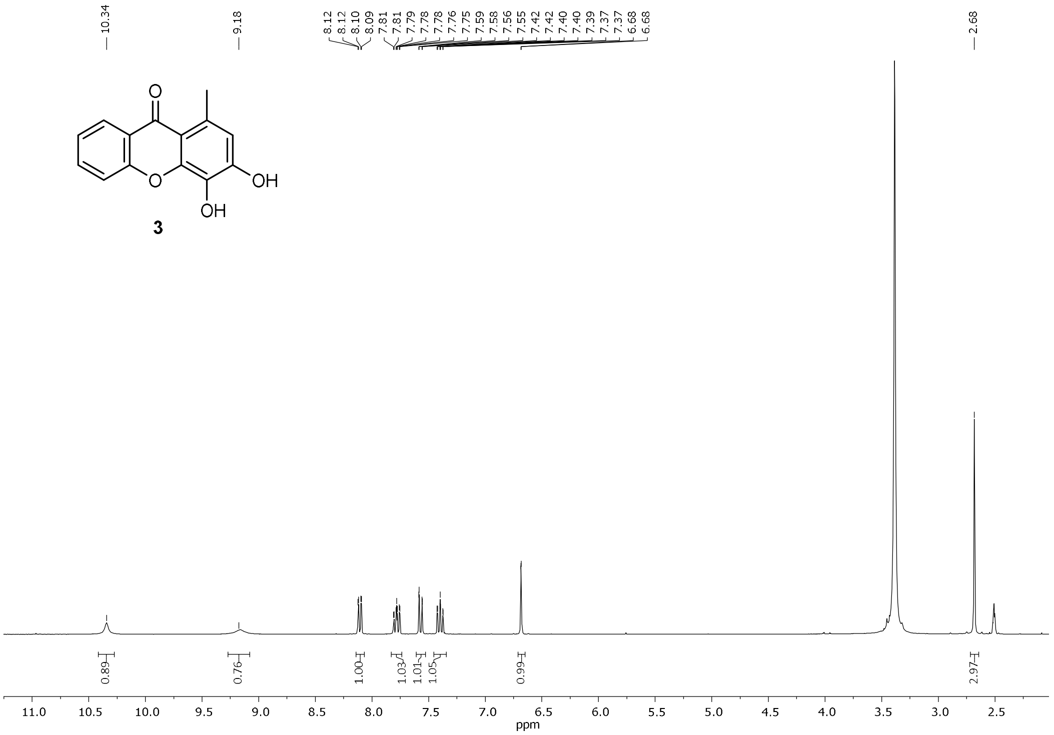

*Figure S1. ^{1}H NMR spectrum of 3,4-dihydroxy-1-methyl-9H-xanthen-9-one (**3**) ($CDCl_3$, 300 MHz).*

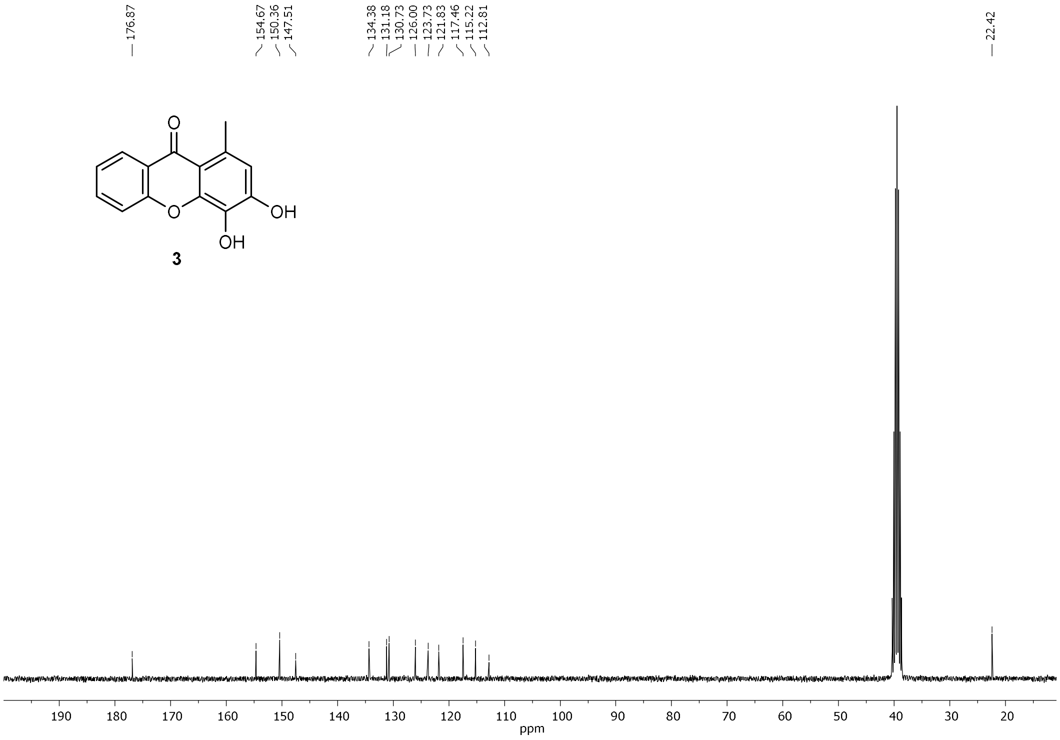

*Figure S2. ^{13}C NMR spectrum of 3,4-dihydroxy-1-methyl-9H-xanthen-9-one (**3**) ($CDCl_3$, 75 MHz).*

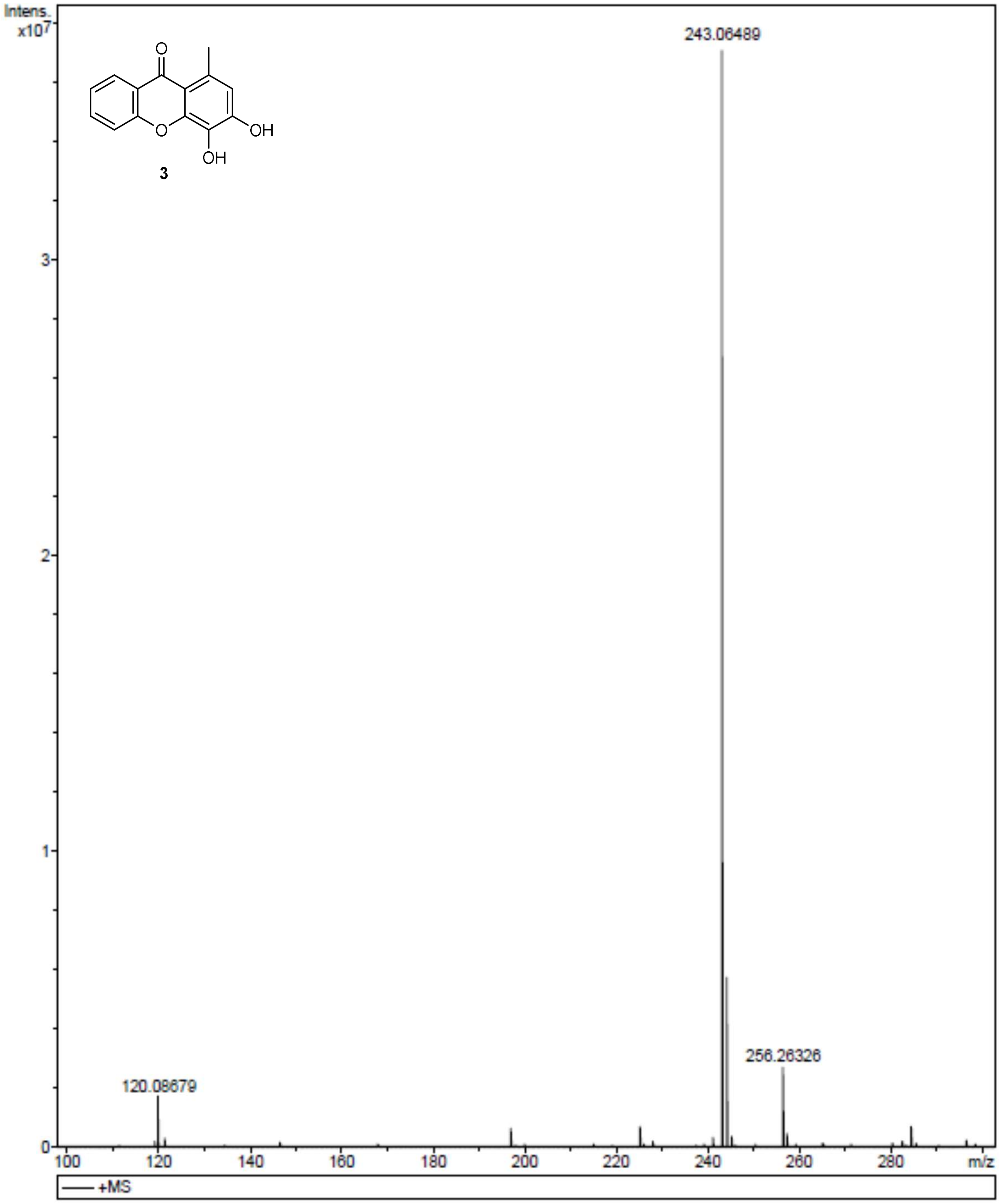

Mass Spectrum Molecular Formula Report

Meas. m/z	#	Formula	Score	m/z	err [mDa]	err [ppm]	mSigma	rdb	e⁻ Conf	N-Rule
243.06489	1	C 14 H 11 O 4	100.00	243.06519	0.29	1.20	11.1	9.5	even	ok

*Figure S3. Electrospray ESI data of 3,4-dihydroxy-1-methyl-9H-xanthen-9-one (**3**).*

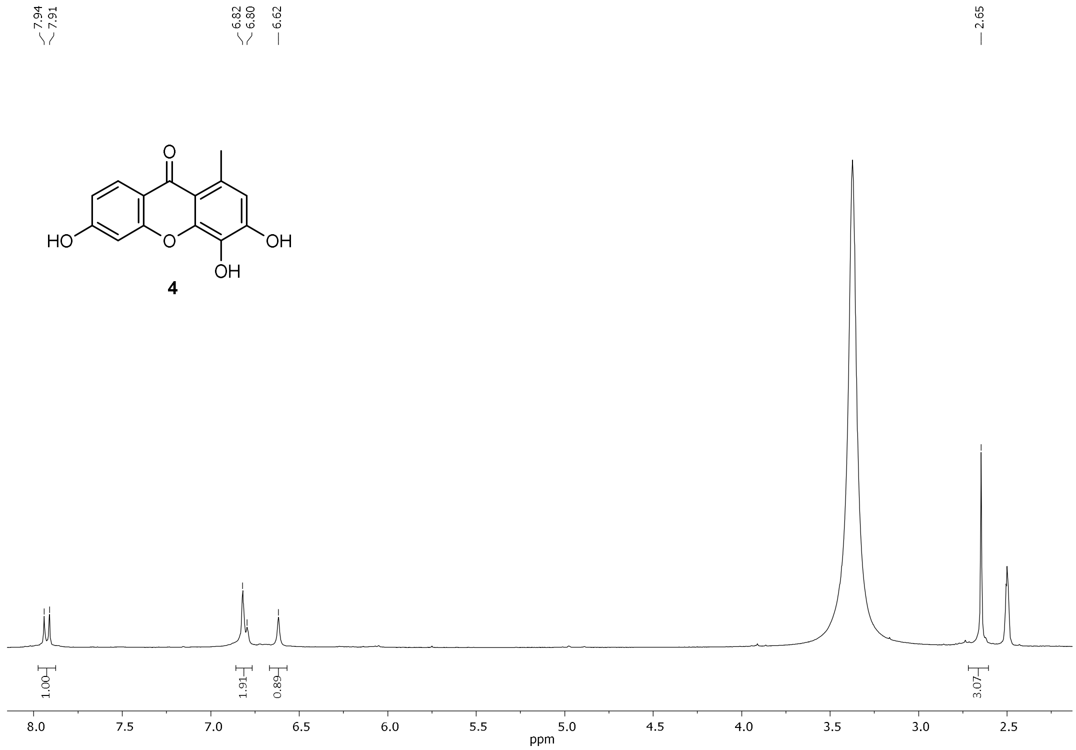

*Figure S4. ^{1}H NMR spectrum of 3,4,6-trihydroxy-1-methyl-9H-xanthen-9-one (**4**) ($CDCl_3$, 300 MHz).*

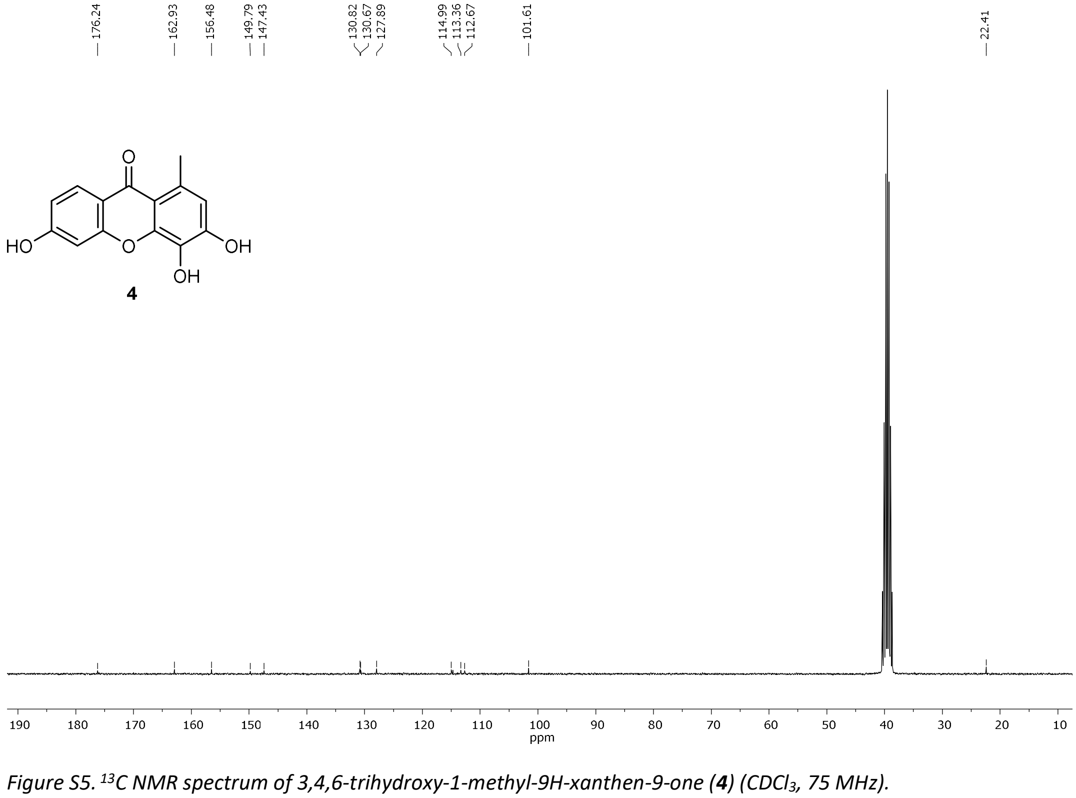

*Figure S5. ^{13}C NMR spectrum of 3,4,6-trihydroxy-1-methyl-9H-xanthen-9-one (**4**) ($CDCl_3$, 75 MHz).*

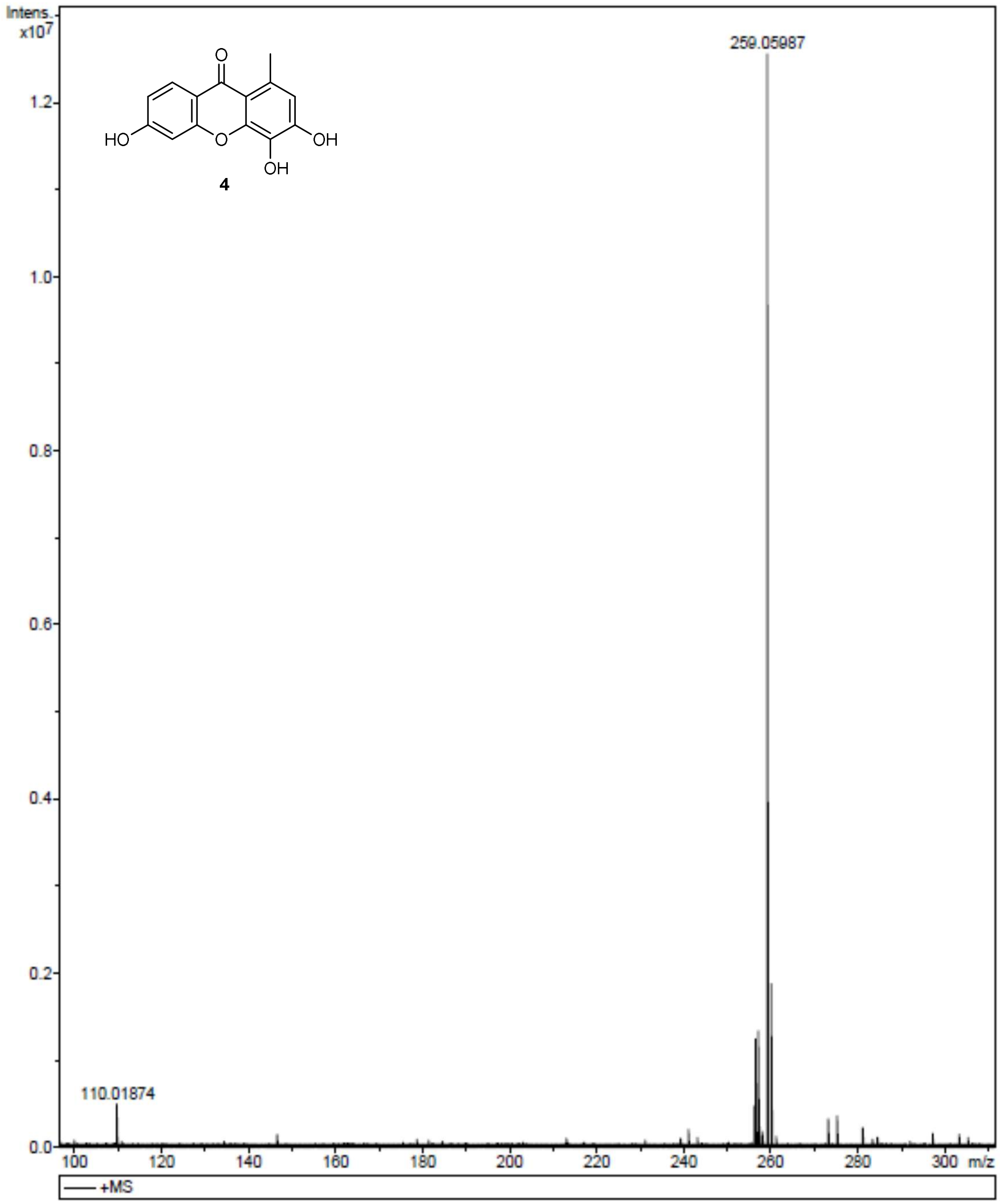

Mass Spectrum Molecular Formula Report

Meas. m/z	#	Formula	Score	m/z	err [mDa]	err [ppm]	mSigma	rdb	e⁻ Conf	N-Rule
259.05987	1	C 14 H 11 O 5	100.00	259.06010	0.23	0.90	12.1	9.5	even	ok

*Figure S6. Electrospray ESI data of 3,4,6-trihydroxy-1-methyl-9H-xanthen-9-one (**4**).*

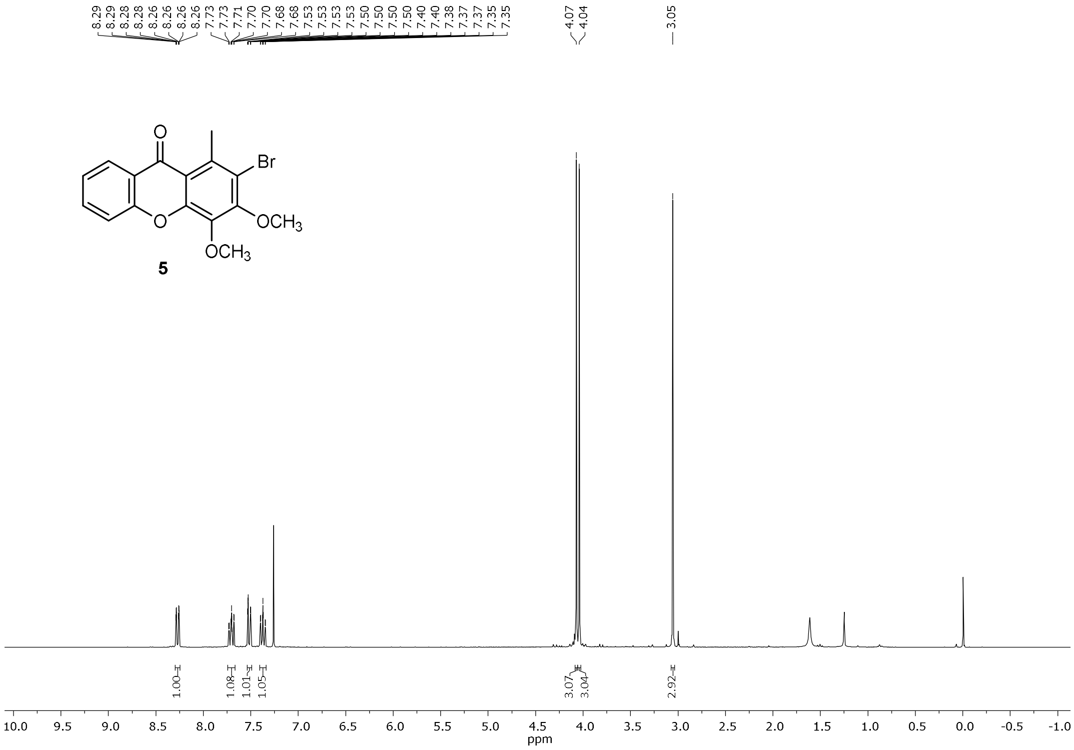

*Figure S7. ^{1}H NMR spectrum of 2-bromo-3,4-dimethoxy-1-methyl-9H-xanthen-9-one (**5**) ($CDCl_3$, 300 MHz).*

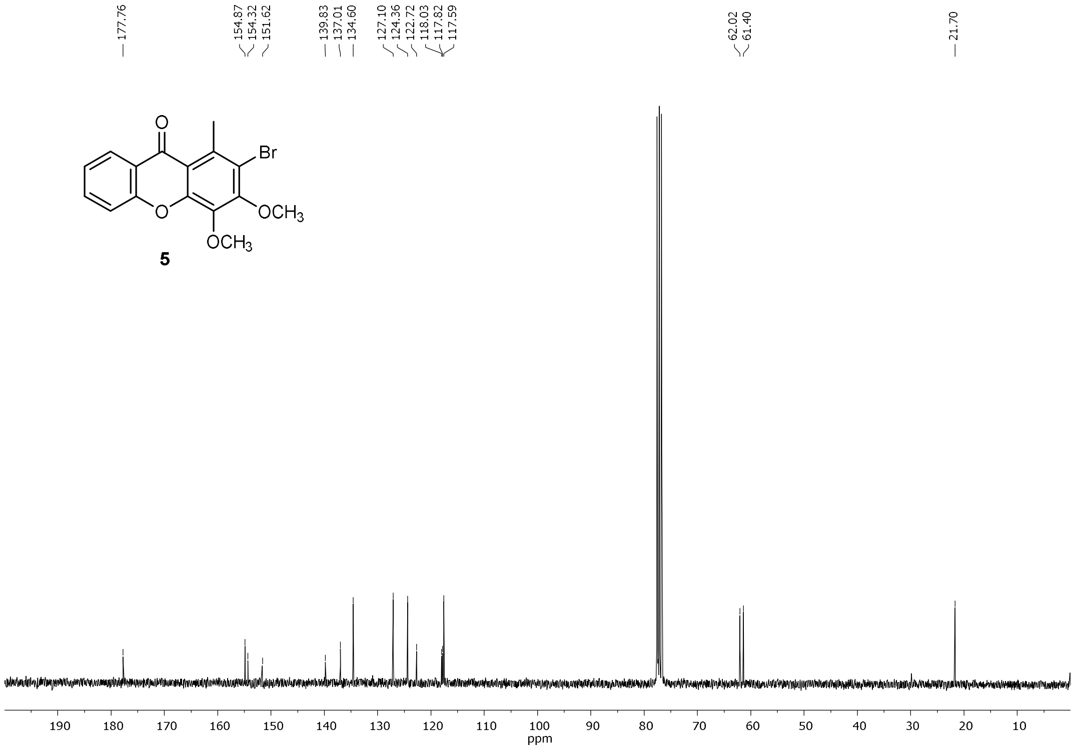

*Figure S8. ^{13}C NMR spectrum of 2-bromo-3,4-dimethoxy-1-methyl-9H-xanthen-9-one (**5**) ($CDCl_3$, 75 MHz).*

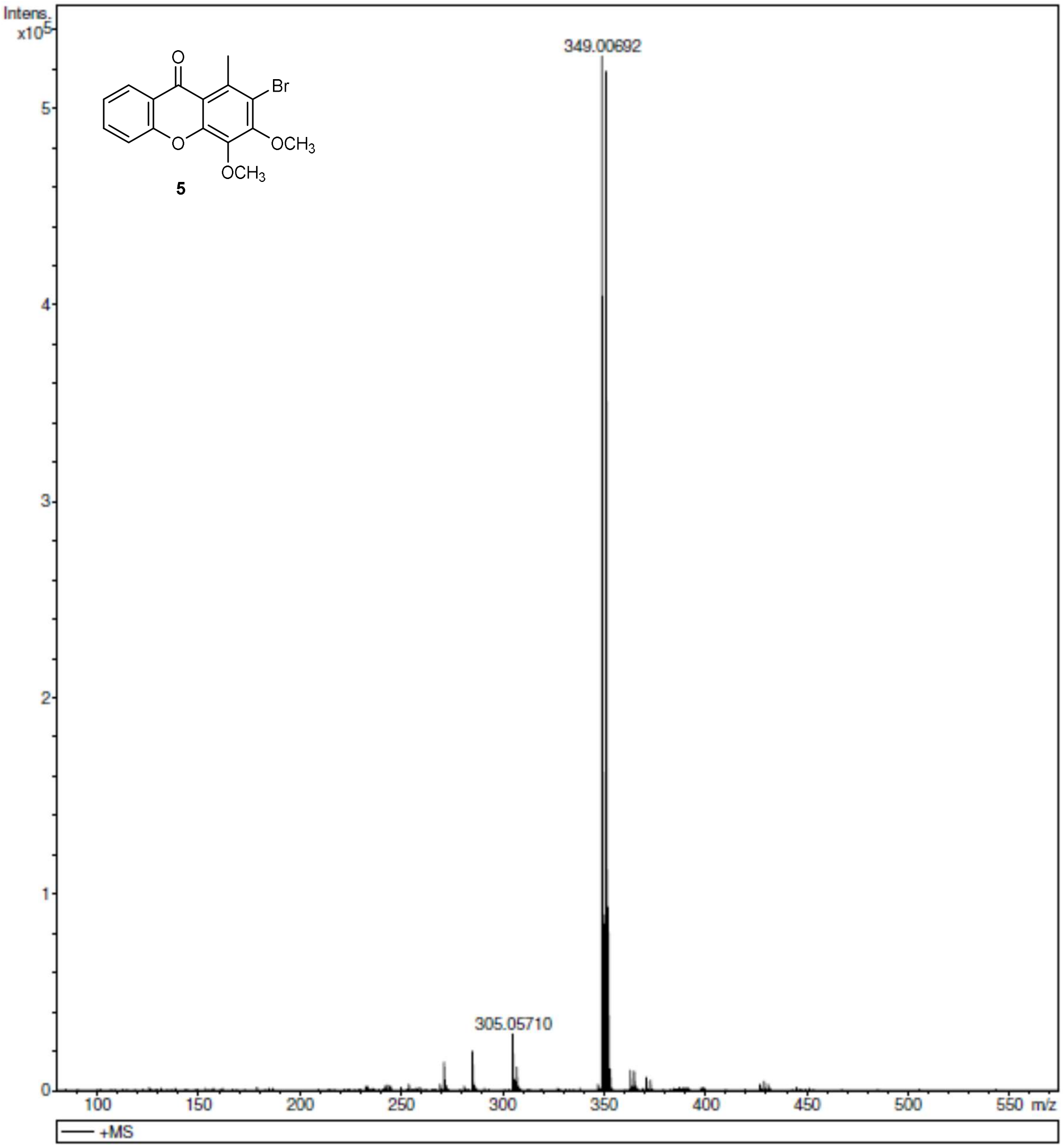

Mass Spectrum Molecular Formula Report

Meas. m/z	#	Formula	Score	m/z	err [mDa]	err [ppm]	mSigma	rdb	e⁻ Conf	N-Rule
349.00692	1	C 16 H 14 Br O 4	100.00	349.00700	0.1	0.2	5.8	9.5	even	ok

*Figure S9. Electrospray ESI data of 2-bromo-3,4-dimethoxy-1-methyl-9H-xanthen-9-one (**5**).*

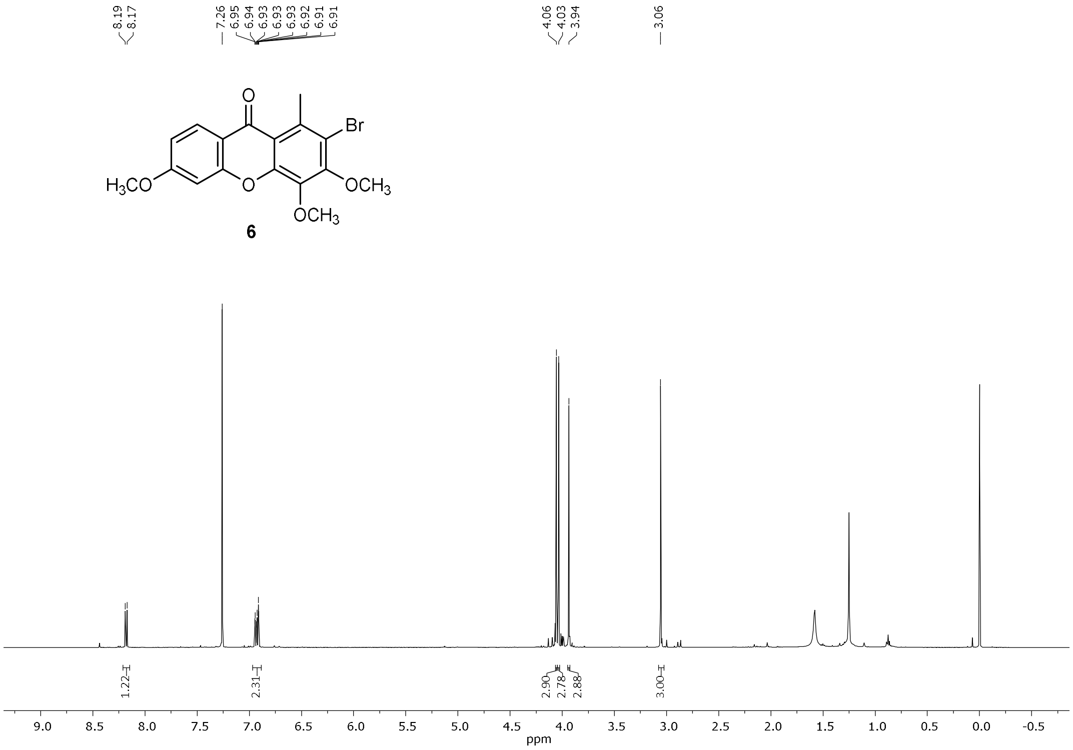

*Figure S10. ¹H NMR spectrum of 2-bromo-3,4,6-trimethoxy-1-methyl-9H-xanthen-9-one (**6**) ($CDCl_3$, 300 MHz).*

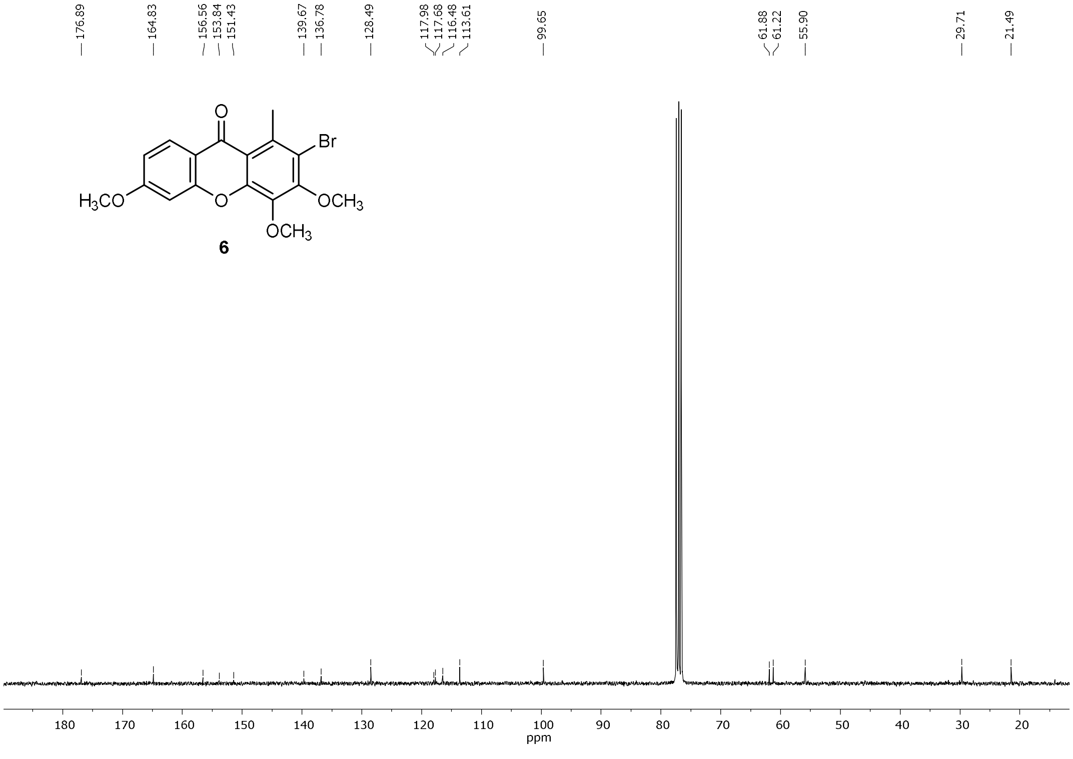

*Figure S11. ¹³C NMR spectrum of 2-bromo-3,4,6-trimethoxy-1-methyl-9H-xanthen-9-one (**6**) ($CDCl_3$, 75 MHz).*

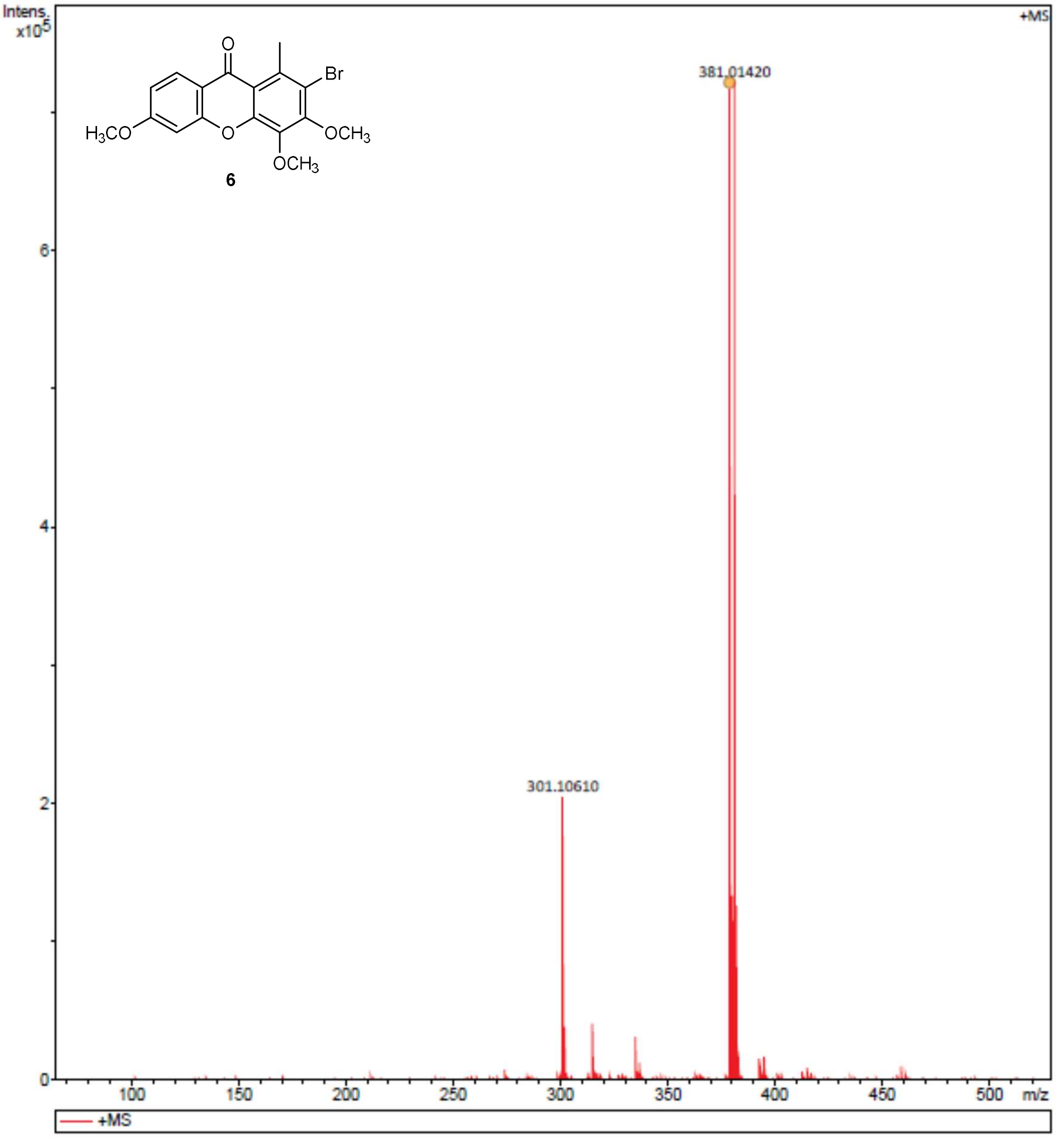

Mass Spectrum Molecular Formula Report

Meas. m/z	#	Ion Formula	m/z	err [ppm]	err [mDa]	mSigma	Score	rdb	e⁻ Conf	N-Rule
379.01614	1	C17H16BrO5	379.01756	3.74	1.42	6.4	100.00	9.5	even	ok

*Figure S12. Electrospray ESI data of 2-bromo-3,4,6-trimethoxy-1-methyl-9H-xanthen-9-one (**6**).*

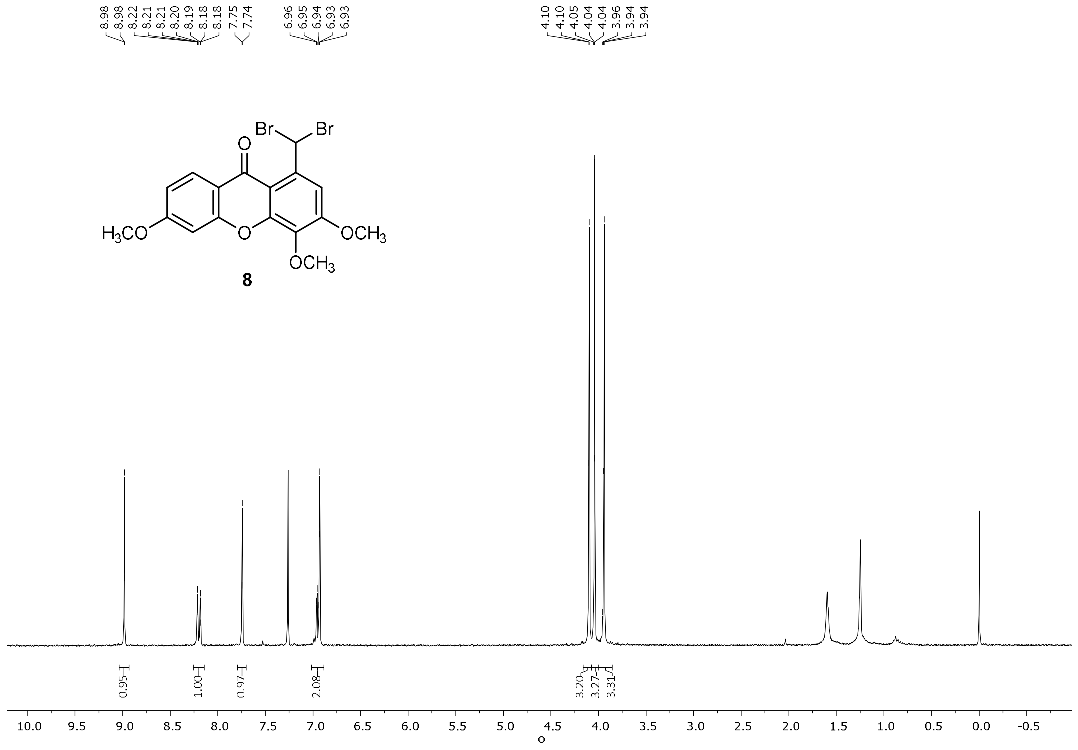

*Figure S13. ^{1}H NMR spectrum of 1-(dibromomethyl)-3,4,6-trimethoxy-9H-xanthen-9-one (**8**) ($CDCl_3$, 300 MHz).*

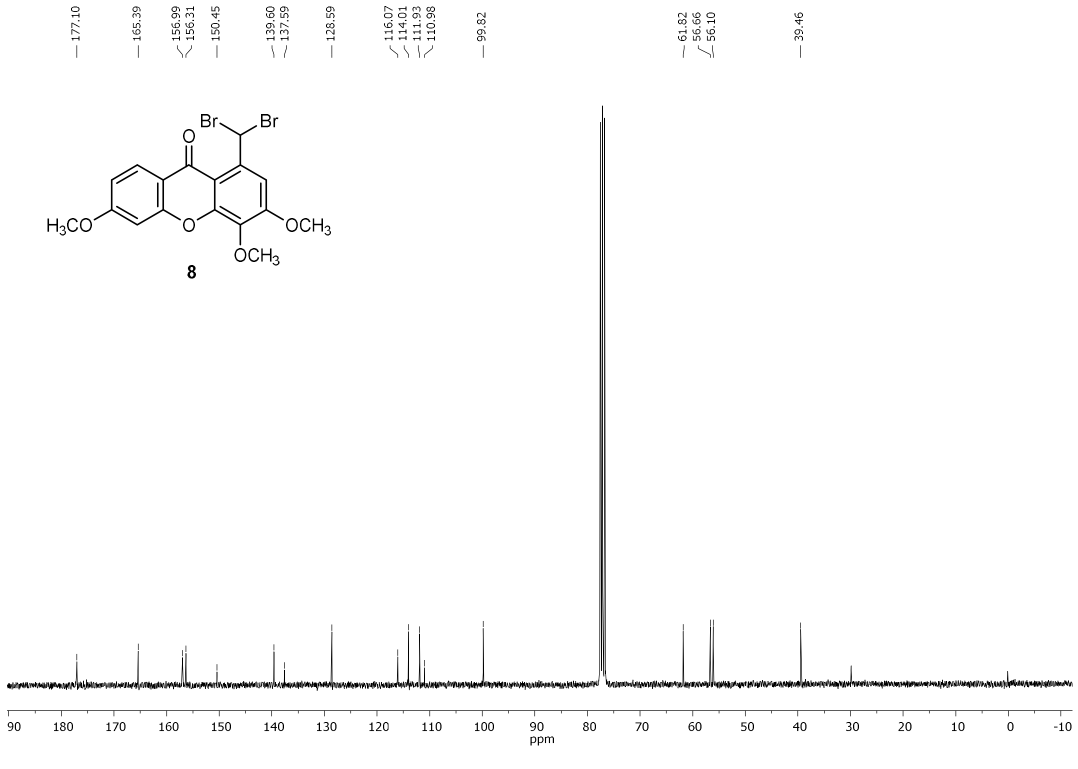

*Figure S14. ^{13}C NMR spectrum of 1-(dibromomethyl)-3,4,6-trimethoxy-9H-xanthen-9-one (**8**) ($CDCl_3$, 75 MHz).*

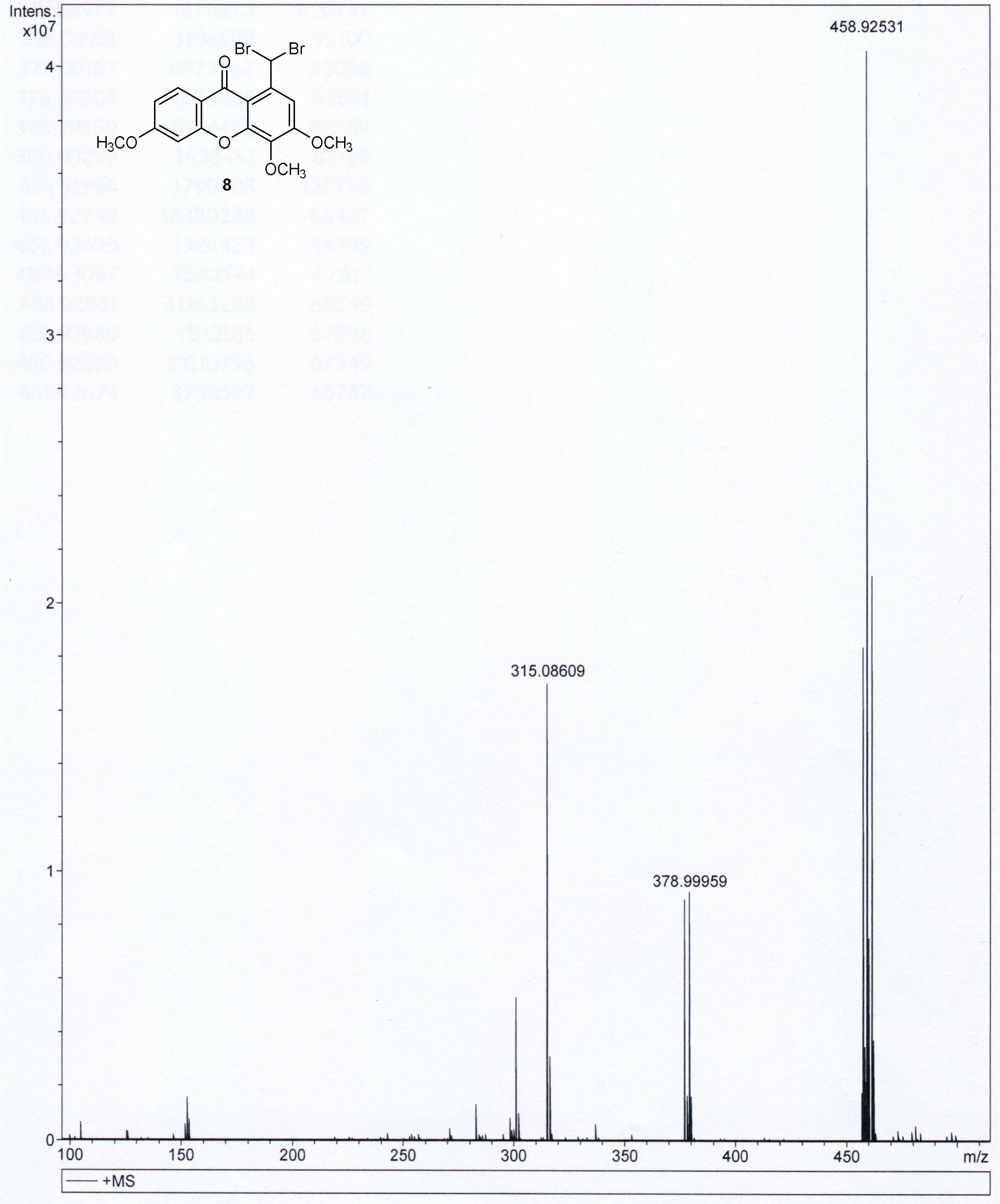

Mass Spectrum Molecular Formula Report

Meas. m/z	#	Formula	Score	m/z	err [mDa]	err [ppm]	mSigma	rdb	e⁻ Conf	N-Rule
456.92748	1	C 17 H 15 Br 2 O 5	100.00	456.92808	0.60	1.31	23.8	9.5	even	ok
	2	C 30 H 2 Br O	0.00	456.92835	0.88	1.92	243.0	29.5	even	ok

*Figure S15. Electrospray ESI data of 1-(dibromomethyl)-3,4,6-trimethoxy-9H-xanthen-9-one (**8**).*

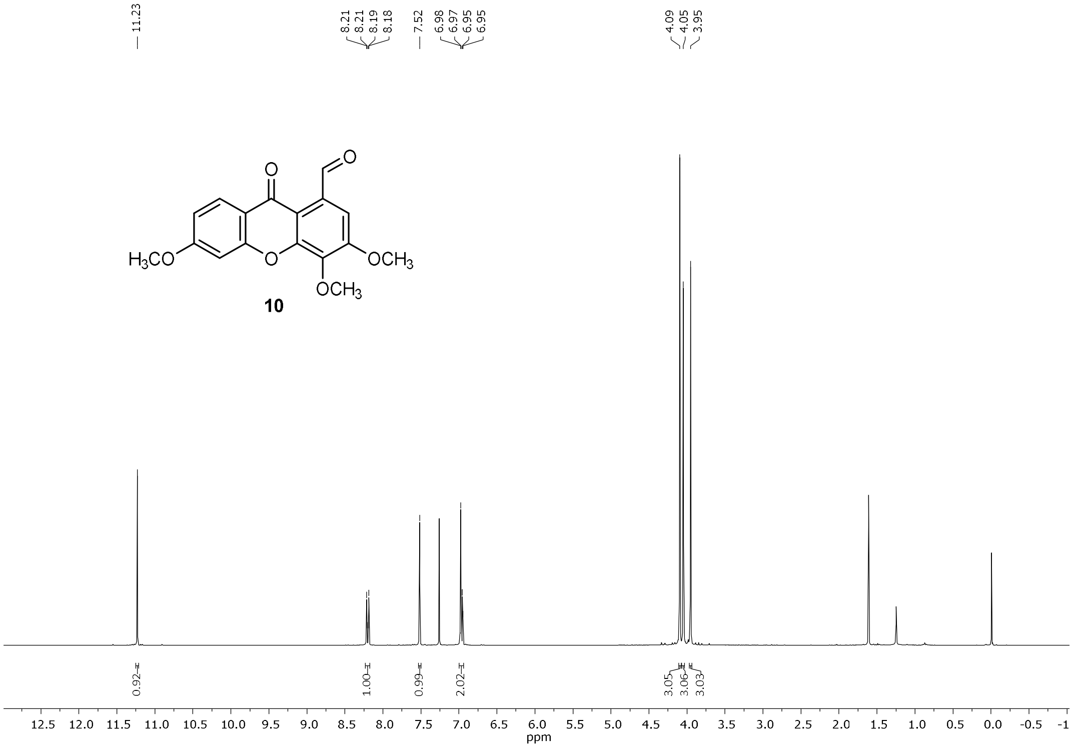

*Figure S16. ^{1}H NMR spectrum of 3,4,6-trimethoxy-9-oxo-9H-xanthene-1-carbaldehyde (**10**) ($CDCl_3$, 300 MHz).*

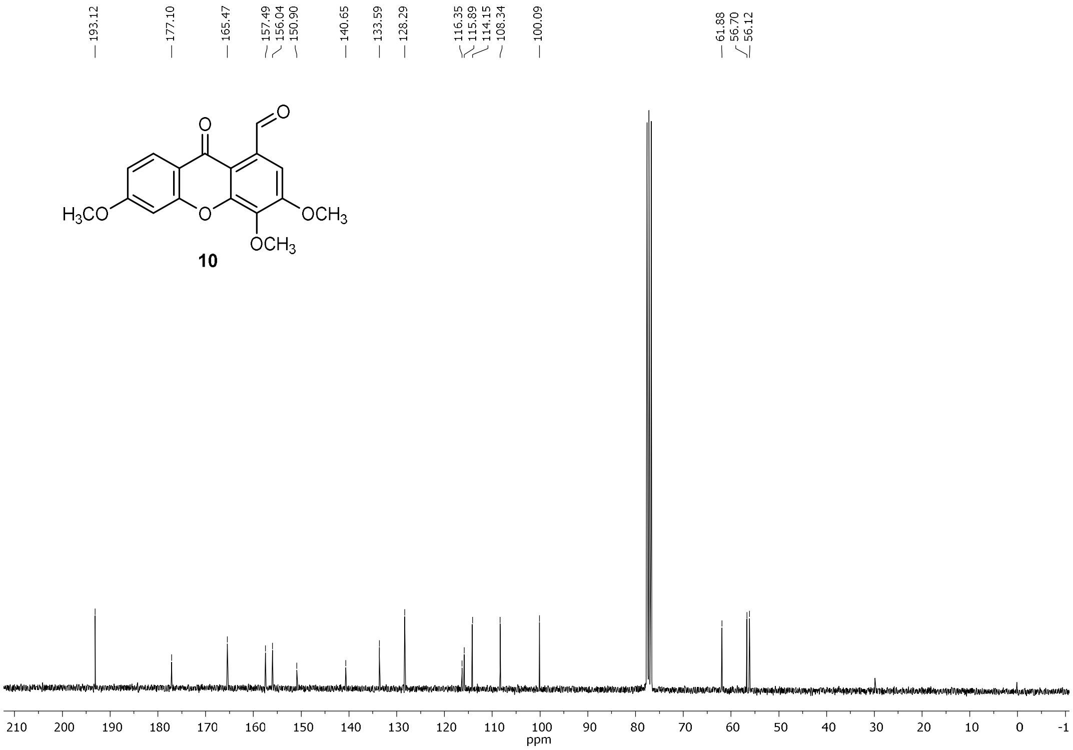

*Figure S17. ^{13}C NMR spectrum of 3,4,6-trimethoxy-9-oxo-9H-xanthene-1-carbaldehyde (**10**) ($CDCl_3$, 75 MHz).*

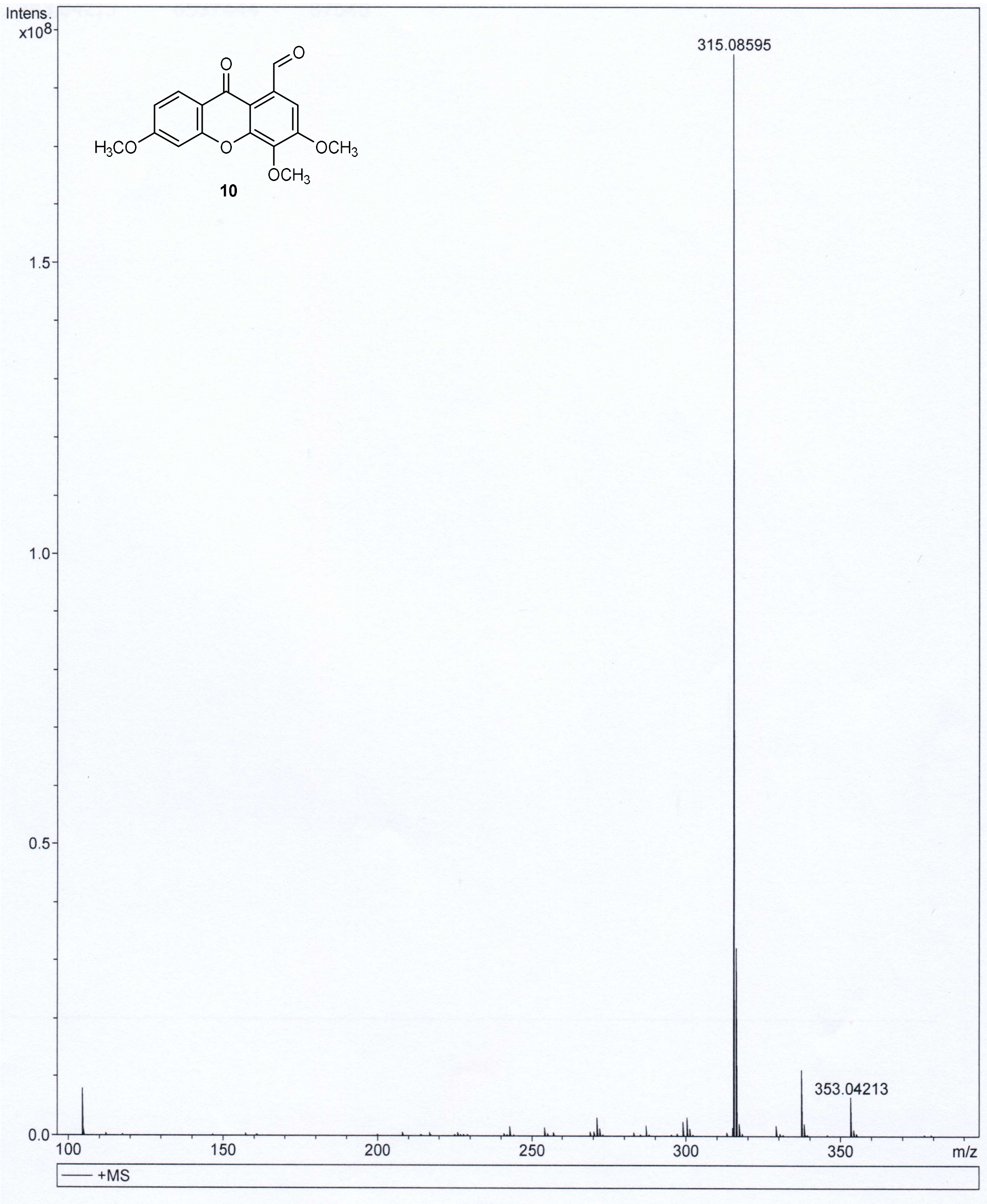

Mass Spectrum Molecular Formula Report

Meas. m/z	#	Formula	Score	m/z	err [mDa]	err [ppm]	mSigma	rdb	e⁻ Conf	N-Rule
315.08595	1	C 17 H 15 O 6	100.00	315.08631	0.36	1.15	17.2	10.5	even	ok

*Figure S18. Electrospray ESI data of 3,4,6-trimethoxy-9-oxo-9H-xanthene-1-carbaldehyde (**10**).*

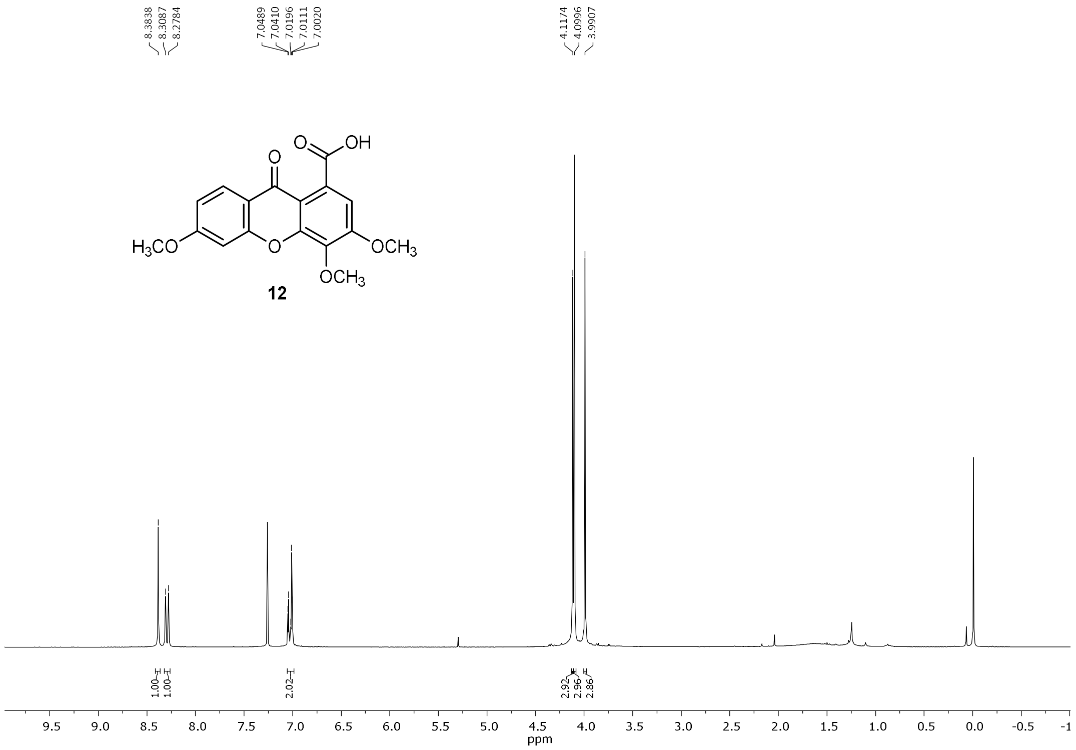

*Figure S19. ^{1}H NMR spectrum of 3,4,6-trimethoxy-9-oxo-9H-xanthene-1-carboxylic acid (**12**) ($CDCl_3$, 300 MHz).*

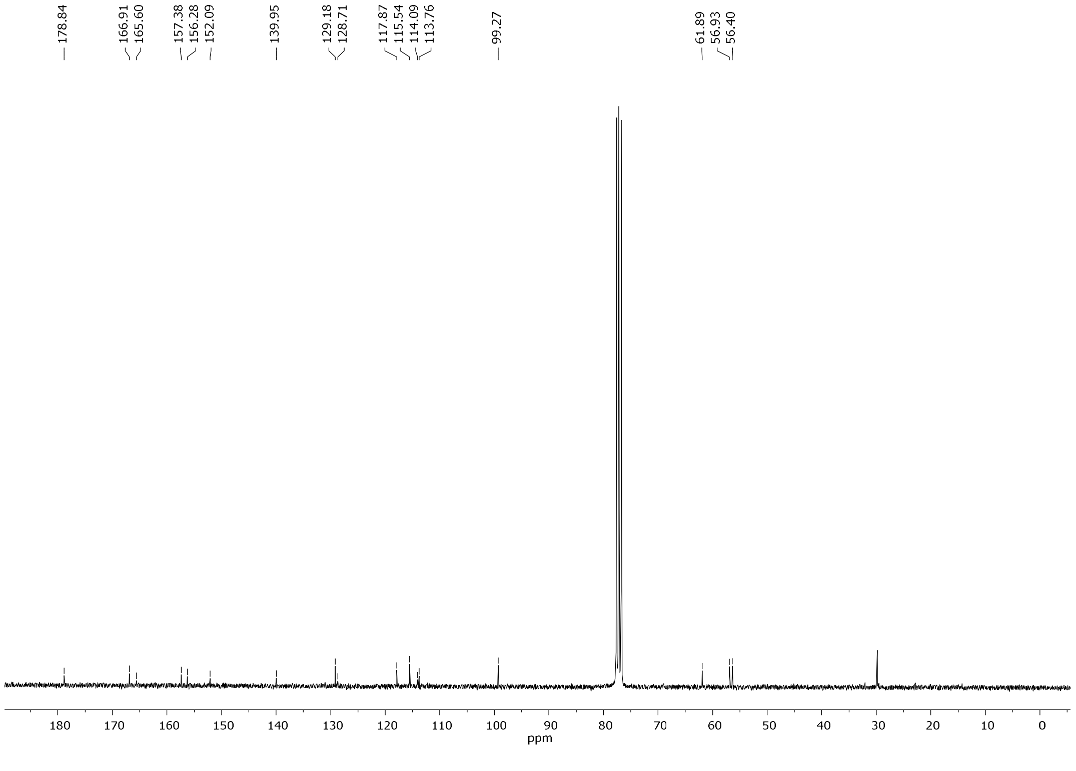

*Figure S20. ^{13}C NMR spectrum of 3,4,6-trimethoxy-9-oxo-9H-xanthene-1-carbaldehyde (**12**) ($CDCl_3$, 75 MHz).*

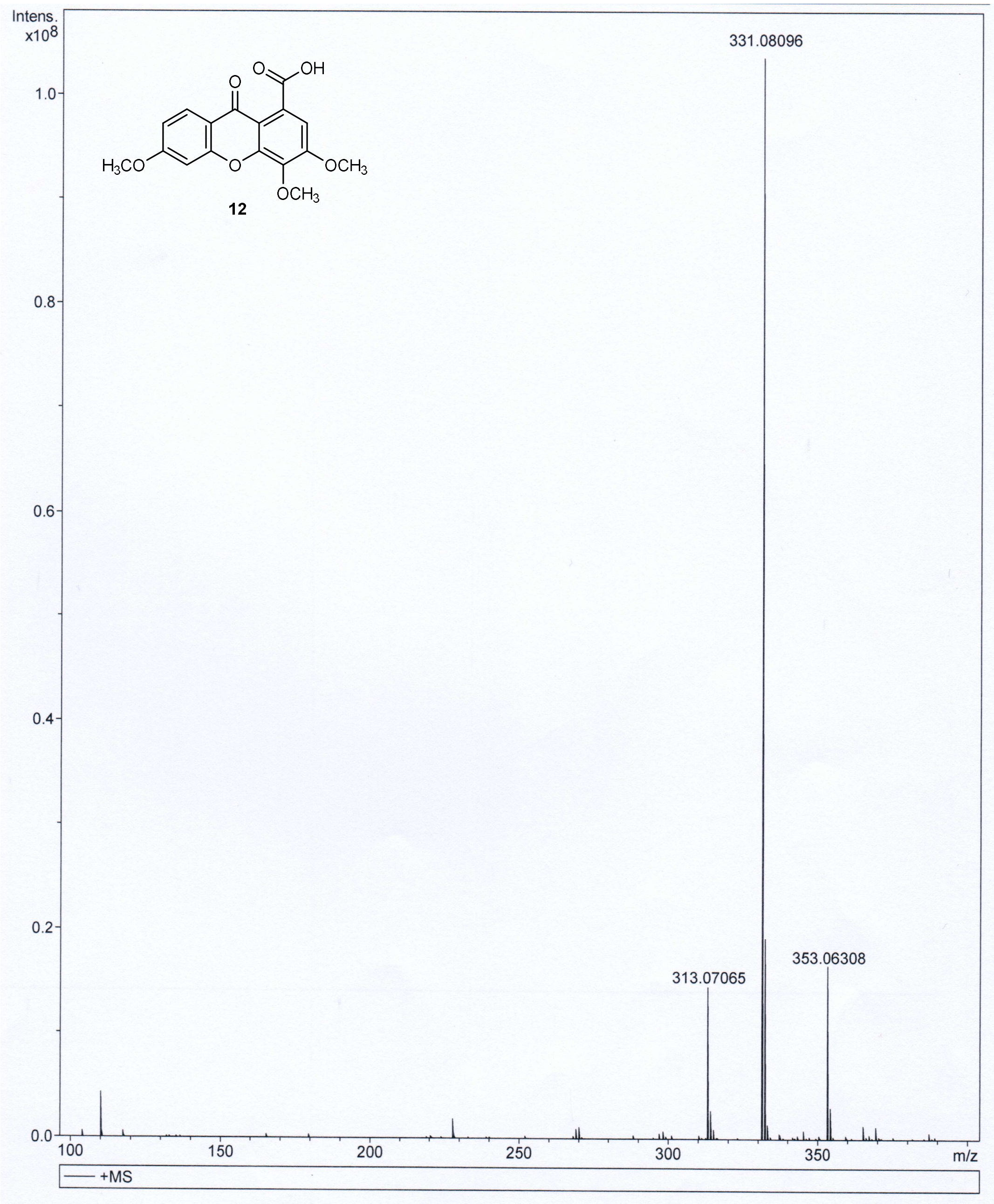

Mass Spectrum Molecular Formula Report

Meas. m/z	#	Formula	Score	m/z	err [mDa]	err [ppm]	mSigma	rdb	e⁻ Conf	N-Rule
331.08096	1	C 17 H 15 O 7	100.00	331.08123	0.27	0.81	17.5	10.5	even	ok

*Figure S21. Electrospray ESI data of 3,4,6-trimethoxy-9-oxo-9H-xanthene-1-carboxylic acid (**12**).*

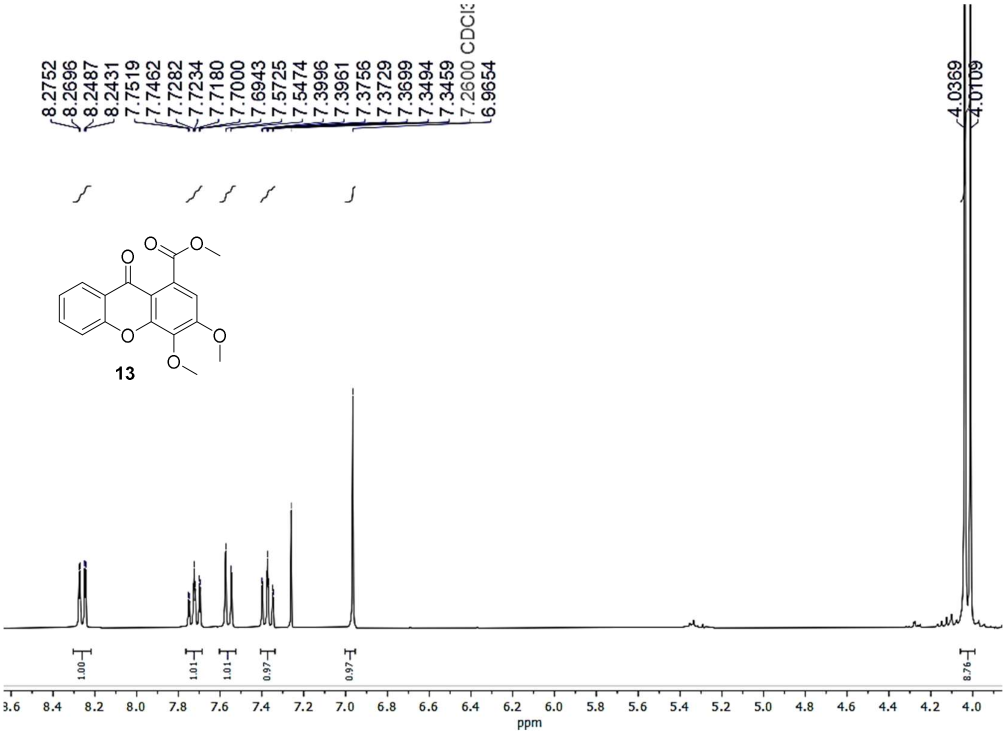

Figure S22. ^{1}H NMR spectrum of 3,4,6-trimethoxy-9-oxo-9H-xanthene-1-carbaldehyde (**13**) ($CDCl_3$, 300 MHz).

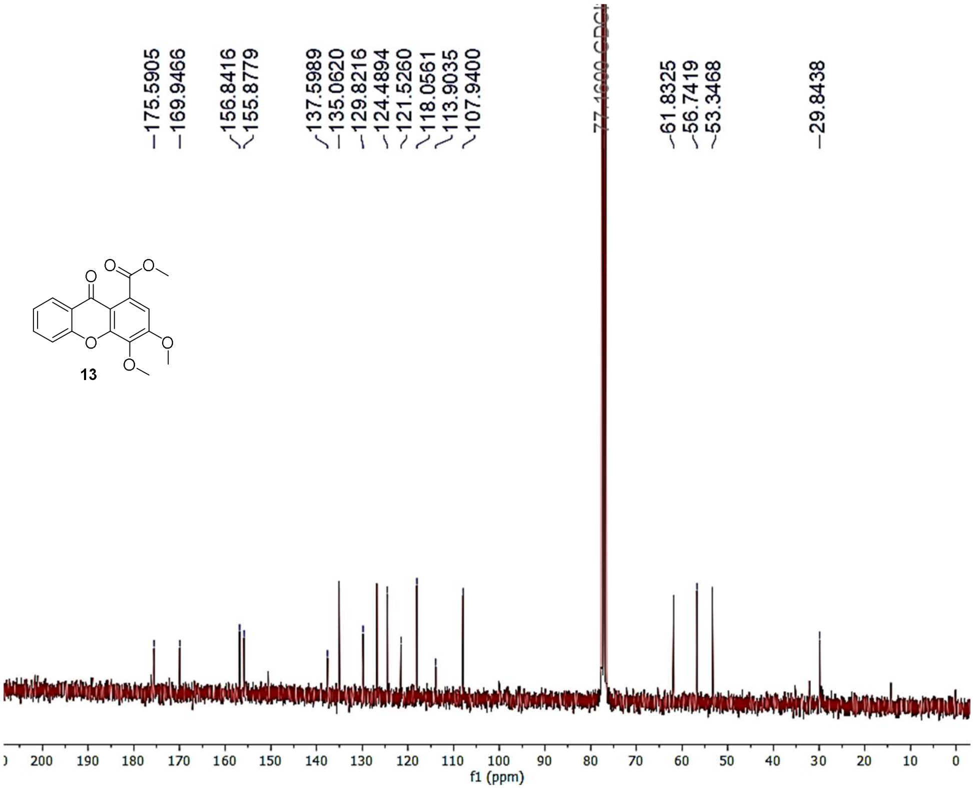

Figure S23. ^{13}C NMR spectrum of 3,4,6-trimethoxy-9-oxo-9H-xanthene-1-carbaldehyde (**13**) ($CDCl_3$, 75 MHz).

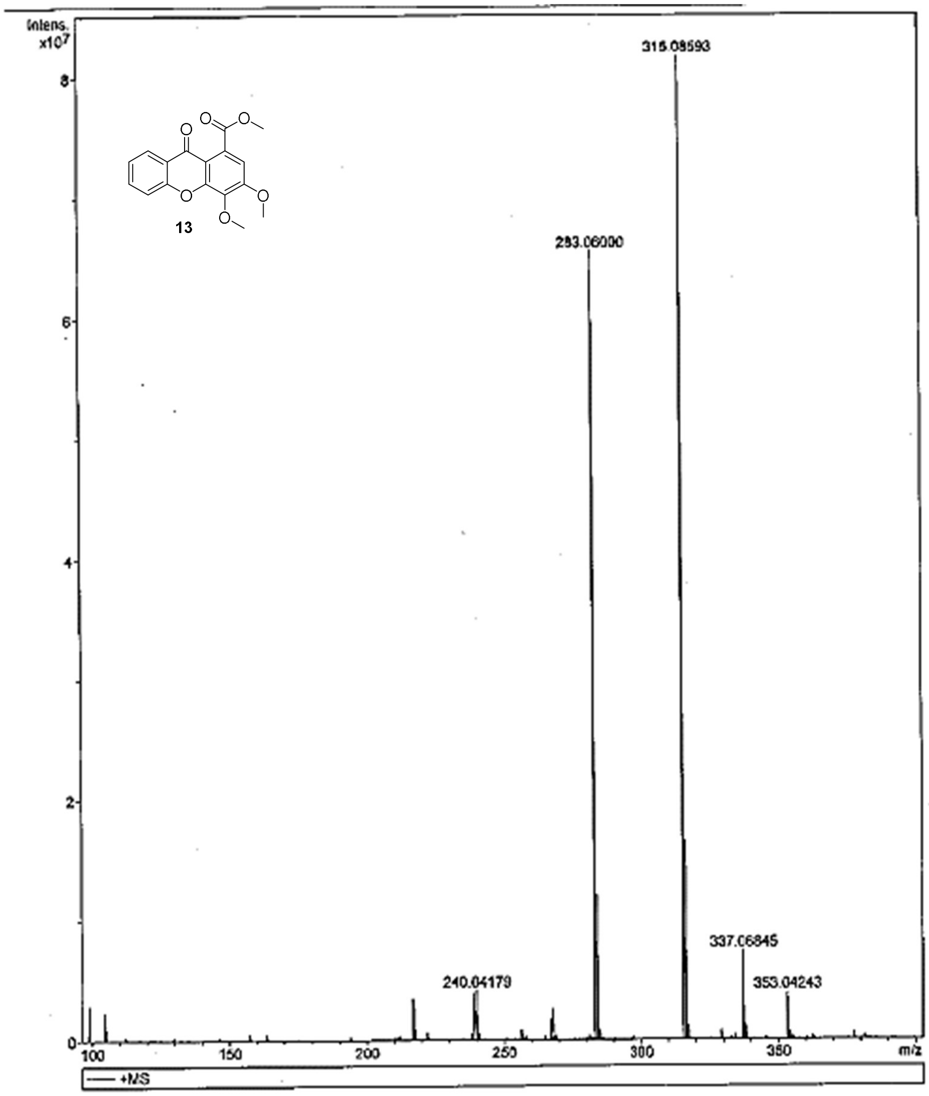

Mass Spectrum Molecular Formula Report

Meas. m/z	#	Formula	Score	m/z	err [mDa]	err [ppm]	mSigma	rdb	e⁻ Conf	N-Rule
283.06000	1	C 16 H 11 O 5	100.00	283.06010	0.10	0.34	15.4	11.5	even	ok
315.08593	1	C 17 H 15 O 6	100.00	315.08631	0.38	1.22	19.2	10.5	even	ok
337.06845	1	C 17 H 14 Na O 6	100.00	337.06826	-0.19	-0.55	107.4	10.5	even	ok
353.04243	1	C 20 H 10 Na O 5	100.00	353.04204	-0.38	-1.08	126.3	15.5	even	ok

*Figure S24. Electrospray ESI data of 3,4,6-trimethoxy-9-oxo-9H-xanthene-1-carbaldehyde (**13**).*

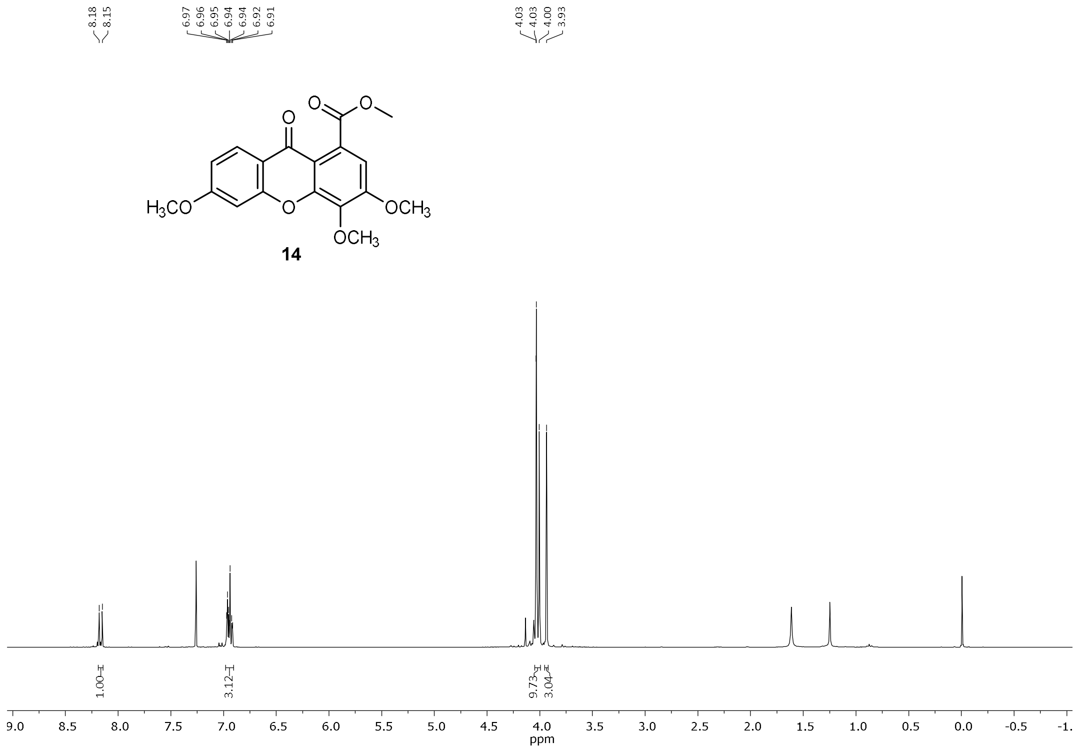

*Figure S25. ^{1}H NMR spectrum of methyl 3,4,6-trimethoxy-9-oxo-9H-xanthene-1-carbaldehyde (**14**) ($CDCl_3$, 300 MHz).*

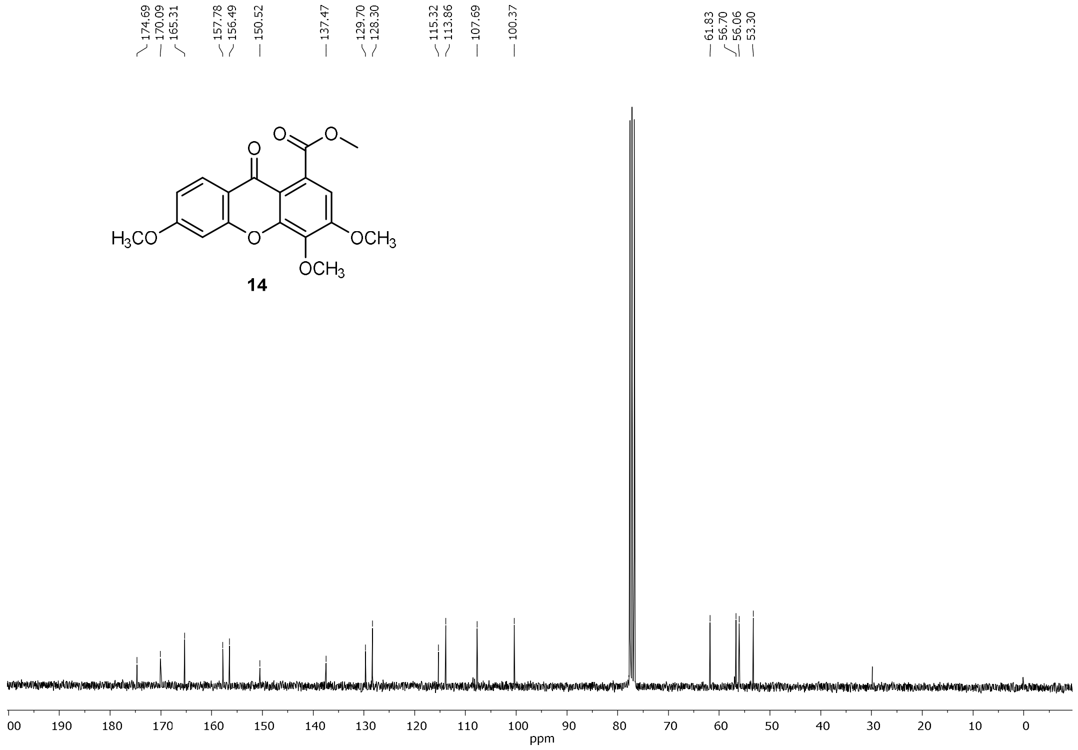

*Figure S26. ^{13}C NMR spectrum of methyl 3,4,6-trimethoxy-9-oxo-9H-xanthene-1-carbaldehyde (**14**) ($CDCl_3$, 75 MHz).*

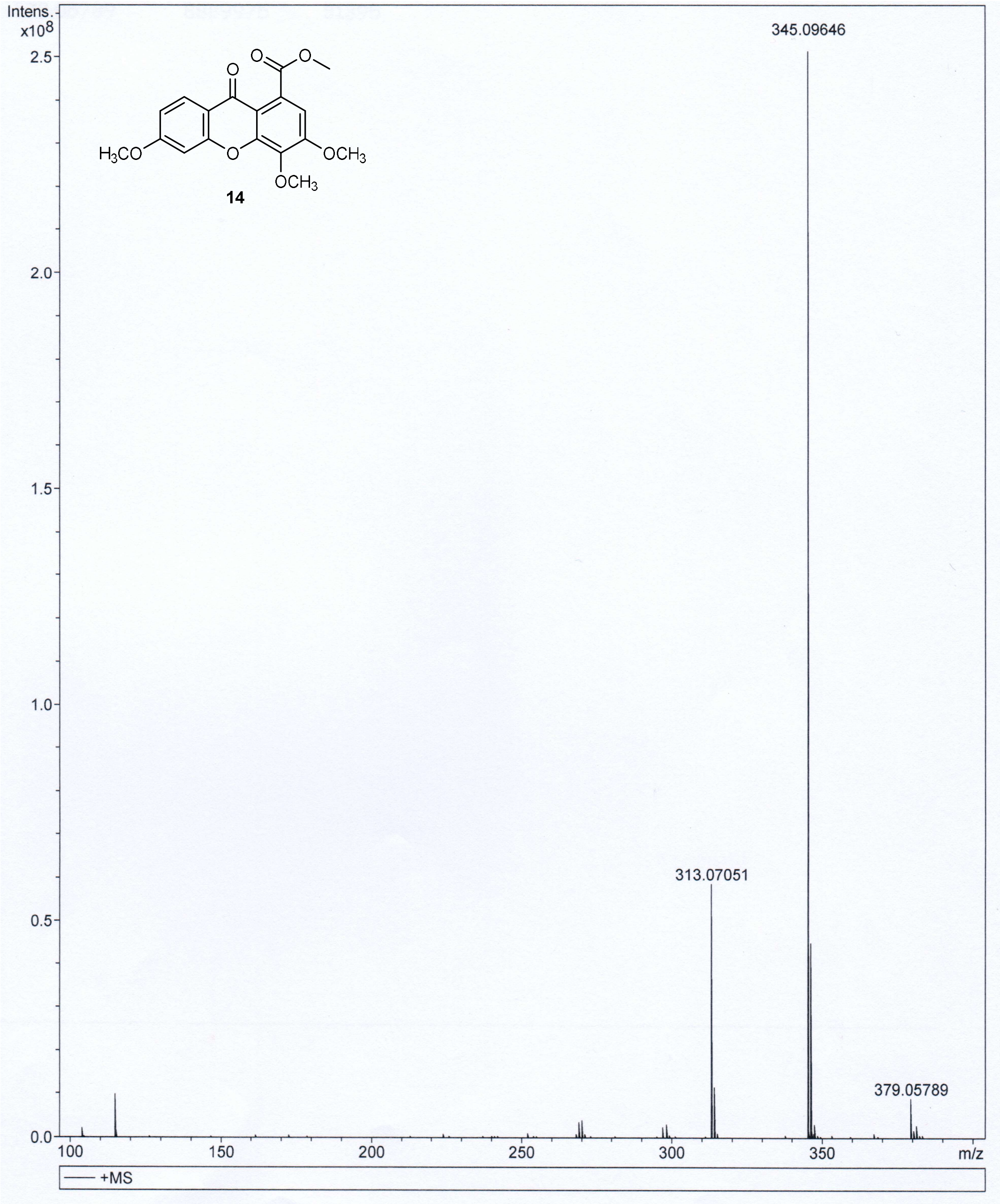

Mass Spectrum Molecular Formula Report

Meas. m/z	#	Formula	Score	m/z	err [mDa]	err [ppm]	mSigma	rdb	e⁻ Conf	N-Rule
345.09646	1	C 18 H 17 O 7	100.00	345.09688	0.42	1.23	21.2	10.5	even	ok

*Figure S27. Electrospray ESI data of methyl 3,4,6-trimethoxy-9-oxo-9H-xanthene-1-carbaldehyde (**14**).*

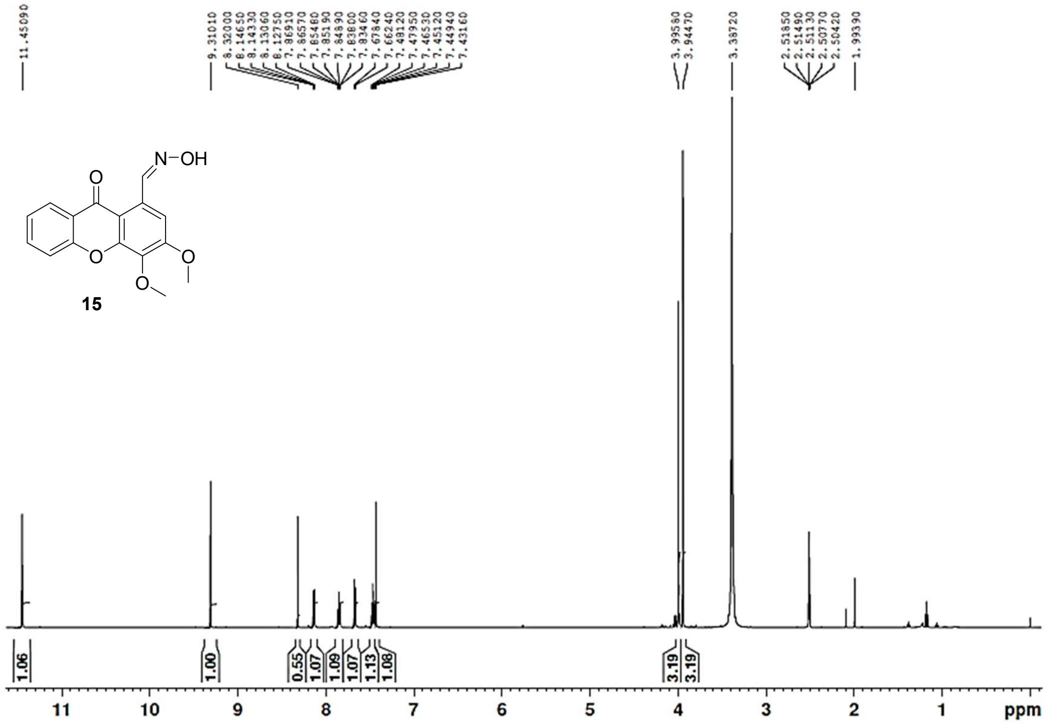

*Figure S28. ¹H NMR spectrum of 3,4,6-trimethoxy-9-oxo-9H-xanthene-1-carbaldehyde (**15**) ($CDCl_3$, 300 MHz).*

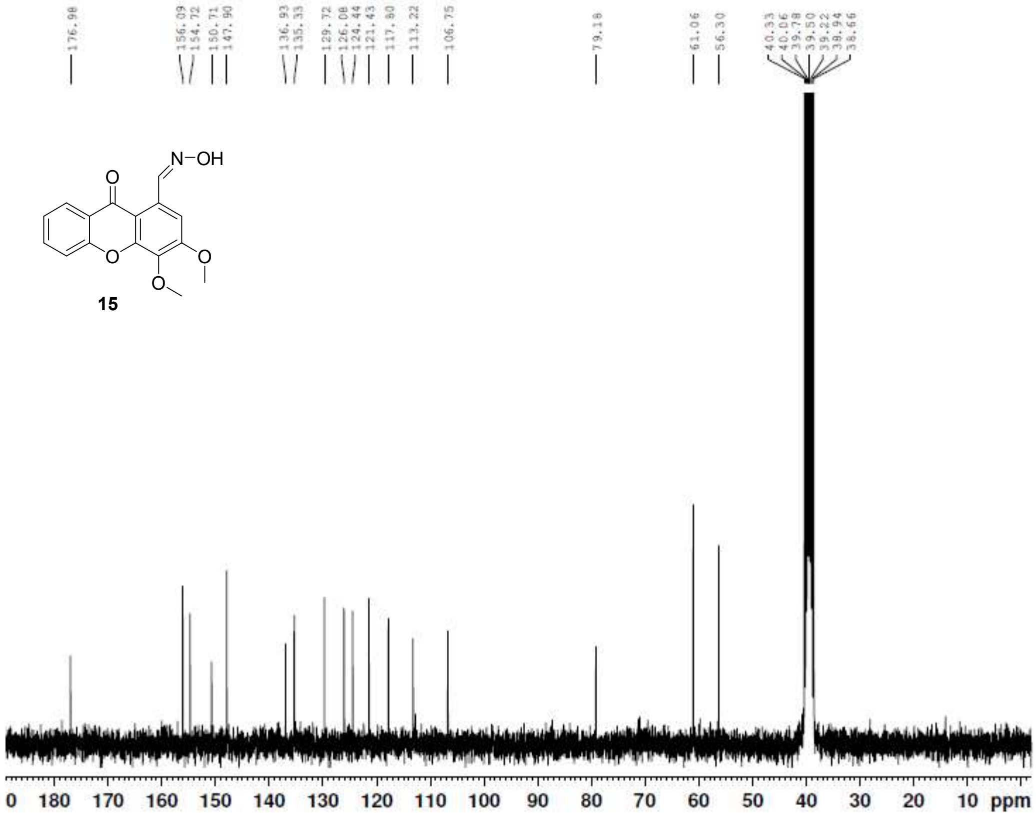

*Figure S29. ¹³C NMR spectrum of 3,4,6-trimethoxy-9-oxo-9H-xanthene-1-carbaldehyde (**15**) ($CDCl_3$, 75 MHz).*

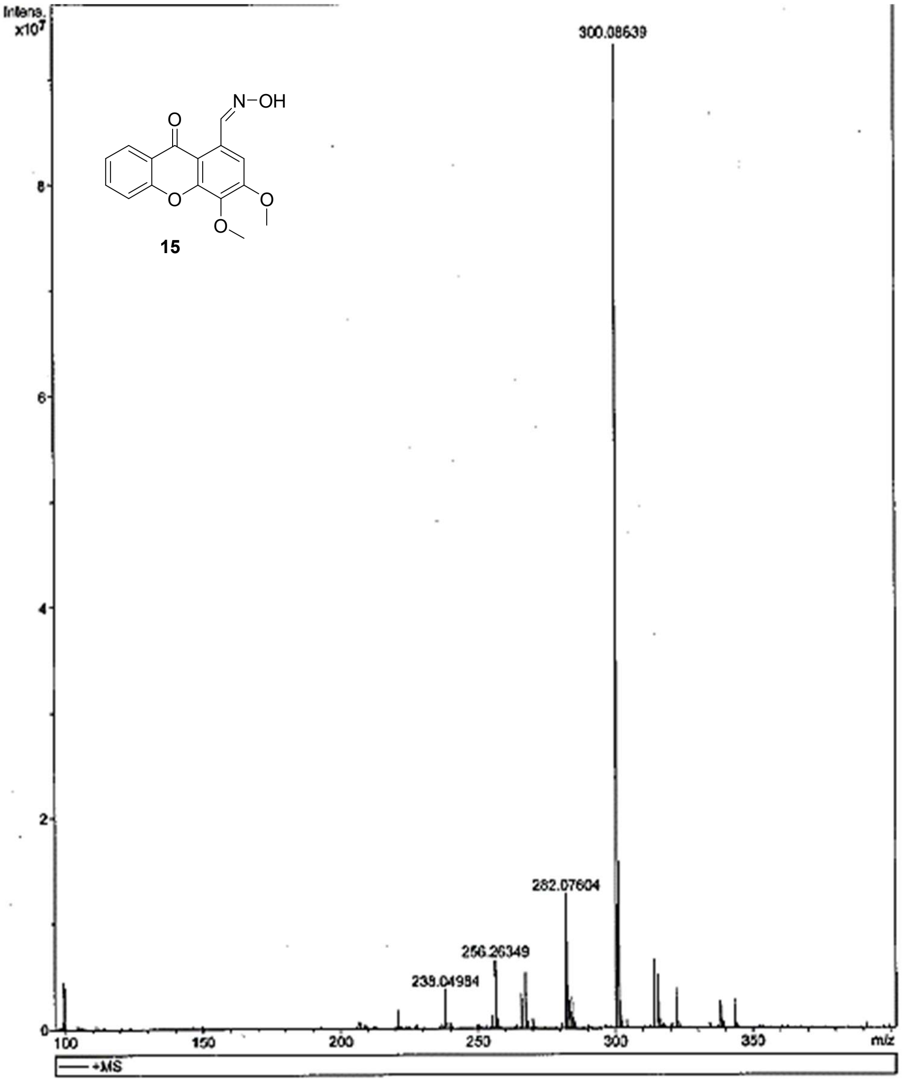

Mass Spectrum Molecular Formula Report

Meas. m/z	#	Formula	Score	m/z	err [mDa]	err [ppm]	mSigma	rdb	e⁻ Conf	N-Rule
300.08639	1	C 16 H 14 N O 5	100.00	300.08665	0.25	0.85	15.1	10.5	even	ok
	2	C 17 H 13 N 2 Na O 2	83.20	300.08692	0.53	1.76	17.9	12.0	odd	ok

*Figure S30. Electrospray ESI data of 3,4,6-trimethoxy-9-oxo-9H-xanthene-1-carbaldehyde (**15**).*
